# A Systematic Review of the Cyclooxygenase-2 (COX-2) Expression in Rectal Cancer Patients Treated with Preoperative Radiotherapy or Radiochemotherapy

**DOI:** 10.3390/jcm10194443

**Published:** 2021-09-27

**Authors:** Monika Berbecka, Alicja Forma, Jacek Baj, Marzena Furtak-Niczyporuk, Ryszard Maciejewski, Robert Sitarz

**Affiliations:** 1 Department of Normal Anatomy, Medical University of Lublin, 20-090 Lublin, Poland; monikapilecka88@gmail.com (M.B.); jacek.baj@umlub.pl (J.B.); maciejewski.r@gmail.com (R.M.); 2 Department of Forensic Medicine, Medical University of Lublin, 20-090 Lublin, Poland; aforma@onet.pl; 3 Department of Public Health, Medical University of Lublin, 20-954 Lublin, Poland; marzenafurtakniczyporuk@umlub.pl; 4 Department of Surgical Oncology, St. John’s Cancer Center, 20-090 Lublin, Poland

**Keywords:** rectal adenocarcinoma, rectal cancer, cyclooxygenase-2, COX-2, radiotherapy, chemoradiotherapy

## Abstract

The main objective of this systematic review is to investigate the expression level of the cyclooxygenase-2 (COX-2) in rectal cancer treated with either preoperative radiotherapy or radiochemotherapy. In addition, we have summarized the effects of preoperative treatment of rectal cancer with regards to the expression levels of COX-2. A systematic literature review was performed in The Cochrane Library, PubMed, Web of Science, and Scopus databases on 1 January 2021 with the usage of the following search string—(cyclooxygenase-2) OR (COX-2) AND (rectal cancer) AND (preoperative radiochemotherapy) OR (preoperative radiotherapy). Among the 176 included in the analysis, only 13 studies were included for data extraction with a total number of 2095 patients. The results of the analysis are based on the articles concerning the expression of COX-2 in rectal cancer among patients treated with preoperative radiotherapy or radiochemotherapy. A COX-2 expression is an early event involved in rectal cancer development. In cases of negative COX-2 expression, radiotherapy and radiochemotherapy might contribute to the reduction of a local recurrence. Therefore, COX-2 may be considered as a biologic factor while selecting patients for more effective, less time-consuming and less expensive preoperative treatment. However, the utility of the administration of COX-2 inhibitors to patients with COX-2 overexpression, in an attempt to improve the patients’ response rate to the neoadjuvant treatment, needs an assessment in further clinical trials.

## 1. Introduction

In the last decades, a key enzyme in prostaglandin synthesis, cyclooxygenase-2 (COX-2), has been considered to play a key role in cancer development and progression [1,2]. Discussion of the COX-2 role in the prediction of response to the preoperative radiotherapy or chemoradiotherapy in rectal cancer requires knowledge of biochemistry and molecular effects of enzymatic activity, as well as an evaluation of the relationship between COX-2 expression and response to ionizing radiation. However, a detailed analysis of prostaglandin biology and mechanisms of interactions is beyond the scope of this review.

Cyclooxygenase (COX) regulates a key step in prostanoid synthesis. COX catalyzes the conversion of membrane phospholipids fraction—arachidonic acid (AA)—into an unstable prostaglandin H2 (PGH2) [3]. This initiates the prostaglandin synthesis trial, which is essential for inflammatory reactions, gastro-intestinal protection, homeostasis and renal hemodynamics. There are two principal isoforms of the COX—the ‘constitutive’ isoform COX-1 and ‘inducible’ isoform COX-2 [4]. These two COX isoforms present a similar catalytic activity; however, they are expressed in slightly different conditions—COX-1 is normal for homeostasis while COX-2 is induced in stress conditions (e.g., in response to injury, exposure to various endotoxins, mitogens and cytokines) and is significantly overexpressed at inflammation sites [5]. Except for the above-mentioned isoforms, a third isoform has been identified—COX-3, which is primarily expressed in the central nervous system and is considered to play a role in the induction of fever and pain processes [6]. According to the old dogma, COX-1 is mainly responsible for the synthesis of the prostaglandins involved in tissue homeostasis, while COX-2 is involved in their production under pathological conditions in particular. In addition, COX-2 is involved in a vast number of cellular processes, including gene expression, cellular differentiation, apoptosis, mitogenesis or neoplasia. COX-2 might be undetectable or present in low quantities in most of the tissues since it is primarily upregulated during inflammation; moreover, its expression might be significantly overexpressed in various cancerous tissues confirming its role in the induction and progression of a wide spectrum of pathological events during inflammation or dysregulated homeostasis [7,8]. The overexpression of COX-2 and COX-2 mRNA is not limited just to colon cancer but can be considered as a common feature in many solid tumors (head and neck squamous cell carcinomas, mesothelioma, hepatocellular carcinoma, gastric cancer, breast cancer and non-small cell lung carcinoma) [9,10,11,12,13,14]. Growth factors, such as epidermal growth factor (EGF), platelet-derived growth factor (PDGF), pro-inflammatory cytokines, tumor necrosis factor (TNF), tumor promoters, bile acids and ultraviolet B irradiation, are involved in the stimulation of COX-2 expression.

A huge emphasis on the role of COX-2 in carcinogenesis was primarily made since the early 1990s when the first epidemiological reports regarding the effectiveness of the regular non-steroidal anti-inflammatory drugs (NSAIDs) in the prevention of colorectal cancer have appeared. It was established that COX-2 promotes pro-tumorigenic activity by several mechanisms, including angiogenesis development and resistance to apoptosis, modulation of host immune surveillance, increased DNA mutagenesis, activated peroxidase activity and xenobiotic carcinogens, which are all involved in the cancer invasiveness [15,16,17,18]. During AA metabolism by COX, also called prostaglandin H (PGH) synthase, several chemicals are metabolized. Sometimes, as a result of chemical metabolism, reactive metabolites of mutagenic and potentially carcinogenic activities can also be created. In addition, the peroxidase activity of COX can convert procarcinogens into carcinogens that are active in the tumor formation, as well as stimulate the conversion of the co-oxidized xenobiotics into mutagens. Several compounds that are obtained during the oxidation of the AA (malondialdehyde) can even form the adducts with the DNA. The demonstrated ability of the COX enzymes to activate environmental carcinogens as well as the pathways of aromatic and heterocyclic amines and polycyclic hydrocarbons is crucial in carcinogenesis.

So far, there are numerous reports describing tumorigenesis induced by the overexpression of COX-2. Liu et al. have observed an intensified COX-2 expression in the epithelial cells of the mammary glands, which was associated with a higher risk of hyperplasia and carcinomatosis [19]. One of the characteristics of colorectal cancer is the excessively high amount of the COX-2 protein or COX mRNA compared to the surrounding non-cancerous mucosa [20,21,22]. As a result, the expression of COX-2 is elevated in up to 90% of colorectal cancers. It has been proved that COX-2 expression is increased in adenoma and carcinoma; the COX-2 expression is higher in larger tumors and deep invasions, but the expression was not reported to be related neither to the tumor stage nor the metastasis. Nevertheless, it was shown that COX-2 expression correlates with tumor grade and stage, overall survival, as well as extent of the angiogenesis [23,24,25,26,27,28].

Recently, a wide range of different phenotypes and molecules (K-ras, p53, EGFR, microsatellite instability) have been studied as potential biological markers and prognostic factors of colorectal cancer. However, data regarding their association with the survival rate of colorectal cancer patients remains slightly inconsistent either considering those factors as insignificant [29,30,31] or contrarily, proving their relevance [32,33,34,35]. The latest research proves that increased COX-2 expression is an early event involved in rectal cancer development; evidence from practical and clinical studies indicates that COX-2—derived prostaglandins play an essential role in inflammation, immune response suppression, apoptosis inhibition, angiogenesis, tumor cell invasion, and metastasis [36,37,38].

Preoperative radiotherapy or chemoradiotherapy followed by a complete tumor resection are both well-established treatment strategies for locally advanced rectal cancer [39,40,41]. Moreover, the preoperative treatment seems to be also a well-suited model to evaluate biological factors. Therefore, several studies that investigated the relationship between COX-2 expression to radiotherapy/chemoradiotherapy and clinicopathologic variables in rectal cancer patients treated with preoperative radio- and chemoradiotherapy were conducted. It was shown that COX-2 inhibition (either by genetic or pharmacological means) might be effective in the inhibition of tumorigenic processes; moreover, several animal and human models showed that NSAIDs and coxibs might be useful in preventing the experimental colon cancer model [42,43,44,45]. Rahman et al. suggested that the usage of COX-2 inhibitors in selected groups of colorectal cancer patients (especially those with significantly upregulated COX-2 and MDR-1 levels) might result in significantly greater tumor regression and greater therapeutic response [46]. The need for more studies regarding the expression of COX-2 in different phases of carcinogenesis is emphasized to develop newer drugs that could potentially inhibit the COX-2 action, leading to greater prevention and therapeutic outcomes [47,48].

In this systematic review, we aim to evaluate whether COX-2 expression might act as a prognostic/predictive factor for overall survival after neoadjuvant radio(chemo)therapy in rectal cancer and whether COX-2 inhibitors might act as additional drugs except for standard therapy. In addition, this paper aims to establish the guidelines for rectal cancer treatment regarding COX-2 expression levels and determine the utility of the assessment of COX-2 expression while predicting the treatment response in patients with rectal cancer who are undergoing preoperative radiotherapy or chemoradiotherapy.

## 2. Guidelines for the Colorectal Cancer Treatment

Colorectal cancer is currently the second leading cause of cancer-related deaths and the third most prevalently diagnosed cancer, according to GLOBOCAN 2020 data [49]. The highest prevalence of colorectal cancer is mainly observed in Europe, New Zealand, Australia and North America [50]. The risk of colorectal cancer increases with age (median age is about 70 years) but other risk factors such as genetic predisposition, improper diet (dietary components deficiencies, consumption of red and processed meat), smoking, inactive lifestyle, precancerous lesions, or other concomitant disorders (diabetes type II, colitis, Crohn’s disease, or hereditary disorders—Lynch syndrome, familial adenomatous polyposis) should also be taken into consideration. Interestingly, NSAIDs intake is considered to be a protective factor against colorectal cancer incidence [51].

Currently, surgery remains the main treatment strategy of rectal cancer with radiation and chemotherapy provided either before or after the surgery. Early rectal cancers and most of the polyps can be removed during a colonoscopy by polypectomy or local excision. More complex types of surgery for rectal cancer include the transanal excision (TAE), transanal endoscopic microsurgery (TEM), low anterior resection (LAR), proctectomy with coloanal anastomosis, abdominoperineal resection (APR), diverting colostomy, or pelvic exenteration. When rectal cancer metastasizes, ablation or embolization might be useful. For rectal cancers, radiation therapy is commonly applied in form of neoadjuvant treatment or intraoperative treatment therapy (IORT). Further, chemotherapy might be provided at different stages of rectal cancer treatment; the most common drugs currently used include 5-fluorouracil, irinotecan, capecitabine, oxaliplatin, and trifluridine and tipiracil [52,53,54,55,56]. Regarding immunotherapy for colorectal cancer, PD-1 inhibitors—pembrolizumab and nivolumab, as well as CTLA-4 inhibitor, ipilimumab—are one of the most prevalently used [57,58,59].

In the last three decades, numerous therapeutic strategies for rectal cancer have evolved. At first, two landmark studies—the National Surgical Adjuvant Breast and Bowel Protocol RO1 trial and the Gastrointestinal Tumor Study Group Protocol 7175 trial—proved the benefits of the adjuvant chemoradiation therapy for local control and long-term survival for patients with advanced carcinoma [60,61]. Several subsequent prospective randomized trials have demonstrated a decrease in a local recurrence and improvement in overall survival as an effect of colorectal cancer neoadjuvant therapy. Currently, preoperative radiotherapy or neoadjuvant chemoradiotherapy (CRT) followed by a complete tumor resection (total mesorectal excision—TME) constitutes a well-established treatment strategy for locally advanced rectal cancer.

Preoperative radiotherapy followed by TME reduces local recurrence rates and is recommended in intermediate cases (cT2, cT3 without threatened factors, some cT4a). In locally advanced, sometimes in non-resectable colorectal cancer cases, preoperative chemoradiotherapy followed by radical surgery even up to 12 weeks later should be provided. Neoadjuvant chemoradiation therapy is considered as a standard treatment aiming to reduce the local recurrence of cancer as well as to improve the marginal overall survival; however, its duration as well as type depends on the recommendations that slightly differ between countries (Table 1) [62,63].

Notwithstanding the benefits, the outcome of patients is still poor with a 5-year disease-free survival estimated at only 66%; therefore, there is an urgent need for further improvements. The main therapeutic problems include the choice of the best standard of preoperative treatment, as well as tumor resistance (especially radio-resistance).

Currently, the main clinical objective is to search for the potential predictors that could identify those patients who would be the most vulnerable to preoperative treatment. The research is focused on the identification of molecular differences between the pre-treatment tumor biopsies of either responders or ‘non-responders’ to treatment. If a prediction of a patient’s response to radiochemotherapy or radiotherapy was possible (in the early phase of treatment), the ‘non-responders’ (radio-resistant) might be selected for the alternative treatment, which aims at improving their response.

## 3. Patients and Methods

A systematic search of the PubMed, Cochrane, Web of Science and Scopus electronic databases was performed on 1 January 2021 with the usage of the following search string—(cyclooxygenase-2) OR (COX-2) AND (rectal cancer) AND (preoperative radiochemotherapy) OR (radiochemotherapy) OR (preoperative radiotherapy) OR (radiotherapy). The above-mentioned terms were chosen in accordance with the Medical Subject Headings. An additional free search term has limited the results only to the articles related to the colorectal cancer. Terms were combined with Boolean operators. A non-mesh search was also performed. The final analysis included studies of any design that were specifically investigating the COX-2 expression assessment in prediction of response for treating rectal cancer with preoperative radiotherapy or chemoradiotherapy. The language of the articles was limited to English while there were no restrictions regarding the year of the publication. All of the analyzed articles were published and peer-reviewed and included only adult patients; the pre-prints were not taken into consideration. The articles with less than 27 patients in a study group as well as the case studies were excluded from the final analysis. Eventually, 13 articles were included in a qualitative synthesis with a time range 2005–2020.

### 3.1. Assessment of Methodological Quality

The methodological quality of the included studies was assessed by M.B. and A.F. All studies were graded according to the EBLIP critical appraisal checklist developed by Glynn L that takes into consideration such variables as study population, data collection, study design, as well as results [64]. An overall validity calculation of ≥75% indicates that the study can be considered as important. Title, abstract and full-text articles of all potentially relevant studies were screened for relevance by two independent (M.B. and A.F.) researchers. Reference lists and all of the articles found on the PubMed, Cochrane, Web of Science and Scopus databases were screened by M.B. and A.F. to ensure that no relevant studies were missed. Discrepancies were discussed and in case of any doubt, they have been resolved through the discussion with a third author—R.S. Preferred Reporting Items for Systematic Reviews and Meta-Analyses (PRISMA) guidelines were followed. Data were arranged in tabular form and qualitatively reviewed.

### 3.2. Outcome Measures

The papers included in the final analysis were analyzed in terms of the relationship between COX-2 expression and the effects of rectal cancer treatment while applying radiotherapy or radiochemotherapy.

## 4. Results

**Figure 1:**
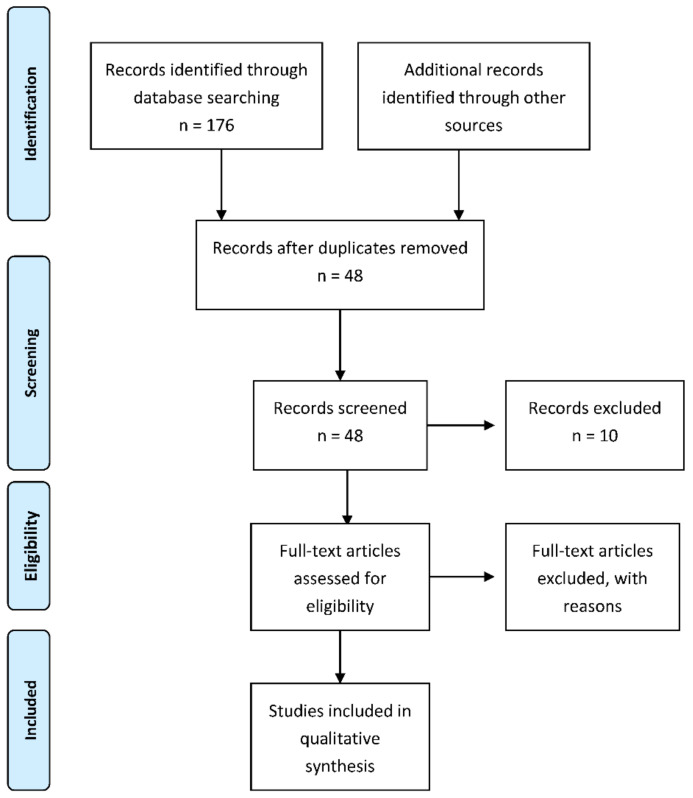
PRISMA flow diagram.

The search revealed 176 articles; after duplicates were removed, 48 articles were screened while 38 of them were assessed for eligibility. The analyzed articles included only those that concerned the studies performed on humans. The time range of the analyzed articles was 2005–2020. Two major inclusion criteria were the expression of COX-2 and either preoperative radiotherapy or preoperative radiochemotherapy applied in rectal cancer patients. Figure 1 depicts the PRISMA flow diagram for study selection. Table 2 depicts general study characteristics.

The search revealed 38 unduplicated articles, among which all of them have undergone a detailed assessment for eligibility providing a total number of 13 articles included in the final analysis. The analysis is based on all of the articles published as full article research (without limitation to language; even though, all of the articles were in English) investigating among others the expression of COX-2 in rectal cancer treated with preoperative radiotherapy or radiochemotherapy. The articles with less than 27 patients were not taken into consideration. No case reports were finally included, six articles were retrospective analyses of COX-2 expression in pretreatment biopsy (paraffin blocks) compared to post-treatment/post-irradiation of COX-2 expression.

The presented systematic review investigated a total of 2095 patients. The studies included in a final analysis were published between 2005 and 2020 and all of them were analyzing the significance of COX-2 expression in either preoperative radiotherapy or radiochemotherapy of rectal cancer, predicting the response of given treatment. In all of the studies, immunohistochemistry was used for COX-2 expression staining. The preparation for quantification of COX-2 was typical—paraffin-embedded tissue from pre-treatment biopsy was prepared and sectioned; then the membrane was blocked and incubated with primary antibodies against COX-2; afterwards, incubation with secondary antibody and detection reagent was performed. In some of the studies, apart from the COX-2 expression, the expression of other molecular markers was performed (p53, p21, p27, Bax, BCL-2, VEGF, APAF-1, CD34, Ki-67, VEGFR-2, EGFR, thymidine phosphorylase and others), which indicates the need for a further search of treatment predictors, including the molecular markers that are involved in cell growth, as well as in the prostanoid and apoptotic pathways [65,66,67,68,69,70,71,72,73,74].

The majority of the tumors (94%) expressed COX-2 only to some degree. COX-2 expression was mainly observed within the cancerous area. Within the tumor cells, the most common immunohistochemical staining pattern of COX-2 was brown, diffuse, granular, cytoplasmic staining. COX-2 expression was also proved to be increased in primary tumors compared with the non-cancerous mucosa (*p* < 0.0001), but no difference was observed between primary tumors and metastases. Heer et al. also investigated the COX-2 expression in relation to apoptosis with similar results—COX-2 expression in non-irradiated surgical specimens was negatively correlated with the apoptosis (*p* = 0.05–0.13); however, it was significantly associated with the decreased level of apoptosis in irradiated tumors (*p* = 0.001) [75]. COX-2 overexpression after radiotherapy was associated with apoptosis resistance and lower levels of radiotherapy-induced apoptosis. It is known that COX-2 induces Bcl-2 (anti-apoptotic protein family) expression, which suppresses radiation-induced cell death, which suggests the COX-2 tumorigenic activity. In Table 2 and Table 3, study characteristics are presented, while Table 4 provides information about the patients’ characteristics.

**Table 2:** Characteristics of the studies regarding COX-2 expression amongst patients with rectal cancer treated with preoperative radiotherapy.

Ref.	Study	Year	Country	Study Design	Analyzed Samples	Total Number of Patients	Treatment Strategy	COX-2 Expression Measurement
[76]	Pachkoria et al.	2005	Sweden	Prospective randomized trial of preoperative radiotherapy	Samples: - distal normal mucosa (*n* = 28) - adjacent normal mucosa (*n* = 108) - primary cancer (*n* = 138) - lymph node metastasis (*n* = 30) - biopsy (*n* = 85)	75 (138—total 75—RTH + surgery 63—surgery alone)	Short-term radiation (radiotherapy 5 × 5 Gy to total dose of 25 Gy) followed by surgery	IHC Western Blott
[75]	de Heer et al.	2007	Netherlands	Retrospective multicenter randomized clinical trial	Archival tumor material	1038 (924—RTH + surgery 927—surgery alone)	Short-term radiation (radiotherapy 5 × 5 Gy to total dose of 25 Gy) followed by TME surgery	IHC
[77]	Giralt et al.	2007	Spain	Retrospective study	Preirridation diagnostic biopsies	34 (81—total 34—radiotherapy 47—radiochemotherapy)	Long-term radiation (radiotherapy: conventional fractionation 1.8 Gy/day to a total dose of 45 Gy; additionally, boost to 50.4 Gy in 8 cases) followed by TME surgery	IHC
[78]	Bouzourene et al.	2008	Switzerland	Retrospective multicenter cohort study	Pretherapeutic tumor biopsies (*n* = 26) and surgical specimens (*n* = 88)	104 (88 specimens 26 pretherapeutic biopsies)	Hyperfractionated radiotherapy (HART) followed by surgery (APR or low anterior resection)	IHC
[79]	Wen et al.	2020	Sweden	Randomized clinical trial	Surgical samples	219 (127—RTH + surgery 92—surgery alone)	Radiotherapy (25 Gy in 5 fractions during a median of 6 days (range, 5–12)) followed by surgery	IHC

**Table 3:** Cyclooxygenase-2 (COX-2) expression in rectal cancer treated with preoperative radiochemotherapy. Study characteristics.

Ref.	Study	Year	Country	Study Design	Analyzed Samples	Total Number of Patients	Treatment Strategy	COX-2 Expression Measurement
[80]	Yeoh et al.	2005	Australia	Retrospective study	Samples obtained from patiets treated with preoperative radiotherapy	28	(1) Radiotherapy (5 × 5Gy) or (2) Radiotherapy (1.8 Gy/day to total 45 Gy; boost 5.4 Gy) (3) Chemotherapy (5-FU at 300 mg/m^2^/day)	IHC
[81]	Smith et al.	2006	Ireland	Retrospective and prospective study	Pretreatment specimens	49	(1) Radiotherapy (to total dose of 45 Gy) (2) Chemotherapy (5-FU at 225 mg/m^2^/day) (3) Surgery	IHC
[77]	Giralt et al.	2007	Spain	Retrospective study	Preirridation diagnostic biopsies	47 (81—total 34—radiotherapy 47—radiochemotherapy)	(1) Radiotherapy (1.8 Gy/day—conventional fractionation to a total dose of 45 Gy; additionally, boost to 50.4 Gy in 8 cases) (2) Simultaneous chemotherapy (5-FU) (3) Surgery—TME	IHC
[82]	Min et al.	2008	Korea	Prospective study	Pretreatment biopsy specimens	30	(1) Radiotherapy (to total dose of 50.4 Gy in 25 fractions) (2) Chemotherapy (5-FU at 425 mg/m^2^/day and Leucovorin 20 mg/m^2^/day during first and fifth week of radiotherapy)	IHC
[83]	Edden et al.	2010	USA	Retrospective study	Preatrement and surgical specimens	152	(1) Radiotherapy (to total dose of 45 Gy; boost to 50.4 Gy); (2) Chemotherapy administrated with radiotherapy (5-FU at 225 mg/m^2^/day or Capecitabine 825 mg 2×/day) (3) Surgery—TME	IHC
[84]	Peng et al.	2016	China	Retrospective study	Pretreatment biopsies	82	(1) Radiotherapy (50 Gy to the rectum as clinical tumor volume, CTV1 and 46 Gy to the region of pelvic lymph node as tumor volume CTV2; in 1.8–2.0 Gy/fraction over a period of 5 weeks (2) Chemotherapy—concurrent with RTH one of two regimens: (1) FOLFOX (fluorouracil 3.0 g/m^2^, CIV lasting for 48 h; calcium folinate 200.0 mg/m^2^, day 1; oxaliplatin 100.0 mg/m^2^, day 1—repeated for 3 weeks (*n* = 6 patients, 7.3%)); (2) XELOX (capecitabine 1000.0 mg/m^2^ bid, days 1–14; oxaliplatin 100.0 mg/m^2^, day 1—repeated for 3 weeks (*n* = 76 patients, 92.7%) (3) Surgery (4) Postoperative adjuvant chemotherapy (XELOX or FOLFOX)	IHC
[85]	Jafarian et al.	2016	Iran	Retrospective cohort study	Pretreatment specimens	55	(1) Radiotherapy—dose of 4500–5040 cGy in 25–28 fractions concurrent with Capecitabine (Xeloda) 825–850 mg/m^2^ dose twice daily (2) Mesorectal resection after 4–6 weeks of neoadjuvant treatment (3) Adjuvant chemotherapy (FOLFOX 4) for 6 months	
[86]	Sole et al.	2016	Spain	Prospective study	Pretreatment endoscopic biopsy and surgical specimens	38	(1) Neoadjuvant chemotherapy (oxaliplatin 85 mg/m^2^ on day 1, leucovorin 200 mg/m^2^ on days 1 and 2, 5-fluorouracil 400 mg/m^2^ on days 1 and 2—over 2 weeks for 2 cycles (FLOFOX-4) (2) Chemoradiotherapy (radiotherapy for 5–6 weeks to a cumulative dose of 45–50.4 Gy (1.8 Gy daily fractions) combined with oral chemotherapy (tegafur at 1200 mg on days 1–4 and 21–25) (3) Surgery	IHC
[87]	Shinto et al.	2020	Japan	Retrospective and prospective study	Pretreatment biopsies	144 (95 in the retrospective study 49 in the prospective study)	In the retrospective study: (1) Short term radiation (to total dose of 20 Gy (5 daily doses of 4 Gy)) (2) Chemotherapy (tegafur-uracil at 400 mg/day for 7 days throughout the period of irradiation) (3) Surgery In the prospective study: (1) Long term radiation (to total dose of 45 Gy (25 daily doses of 1.8 Gy) (2) Chemotherapy (S-1 at 80 mg/day (BSA below 1.25 m^2^), 100 mg/day (BSA 1.25 to less than 1.5 m^2^), 120 mg/day (BSA 1.5 m^2^ or above) and irinotecan at 80 mg/m^2^) (3) Surgery	IHC

**Table 4:** Patients characteristics in the studied articles.

Ref.	Study	Total Number of Patients	Male	Female	Median Age	Age Range
[75]	de Heer et al.	1038 (924—RTH + surgery, 927—surgery alone)	573	324	65	26–88
[78]	Bouzourene et al.	104	ND	ND	63	28–85
[76]	Pachkoria et al.	75	40	23	67	36–85
[81]	Smith et al.	49	31	18	ND	ND
[77]	Giralt et al.	81	54	27	64.8	34–92
[83]	Edden et al.	152	77	75	58.1	31–82
[80]	Yeoh et al.	28	21	7	ND	ND
[82]	Min et al.	30	26	4	48.0	31–69
[87]	Shinto et al.	144	100	44	61,8	ND
[79]	Wen et al.	219 (127—RTH+ surgery, 92—surgery alone)	ND	ND	ND	ND
[84]	Peng et al.	82	57	25	57	15–75
[86]	Sole et al.	38	27	11	62	43–77
[85]	Jafarian et al.	55	27	18	52	18–87

Abbreviations: ND—no data, RTH—radiotherapy.

Only the research by de Heer et al. has found a significant association (*p* = 0.06) between the COX-2 expression and grading; a poor grade of differentiation was associated with high COX-2 expression levels in pretreatment specimens; a high level of COX-2 expression was more often observed both in irradiated and non-irradiated adenocarcinomas [75]. Shinto et al. also proved that low expression of COX-2 was an independent parameter that influenced tumor regression grade (TRG) [87]. According to the results of 75% of the analyzed studies, there is no significant correlation between COX-2 staining and age, gender, tumor downstaging, pT, pN, vascular invasion, tumor necrosis, Duke’s stage, number of tumors and further complications. A relationship of the COX-2 expression in relation to the effects of treatment has been widely measured in numerous articles.

Firstly, weak COX-2 expression was associated with the lower rate of local recurrence after radiation in a study by Pachkoria et al. (*p* = 0.02); Heer et al. proved a correlation with a significantly higher rate of distant recurrences (*p*= 0.005) but not with the local recurrence [75,76]. Wen et al. also showed that patients with high expression of COX-2 were more prone to suffer from tumor recurrence [79]. The possible explanation of COX-2 correlated recurrence is the ability to alternate in the invasive and metastatic potential of cancer cells. Induction of excessive prostaglandin production awakes cell surface glycosyltransferases and type 1 sialyl Lewis antigens, leading to the enhanced tumor cell adhesion to the endothelial cells. An increased prostaglandin production has an immunosuppressive effect, which promotes cells’ escape from the host antitumor response and metastasis [88]. COX-2 is an immediate-early response gene [89]. Secondly, research conducted by Heer et al., shows that tumors with high levels of COX-2 expression after radiotherapy are significantly associated with poor disease-free survival (*p* = 0.004) as well as poor overall survival (*p*= 0.006) [75]. In addition, Bouzourene has shown that disease-free survival (DFS) is longer in patients who are radiosensitive compared to non-responders (*p* = 0.03) [78]. Wen et al. also proved that the patients with high COX-2 expression levels in biopsy samples tended to have worse OS and DFS, with or without RTH [79]. Moreover, Min, Smith, Edden and Jafarian et al. emphasize in their studies that COX-2 overexpression in pretreatment biopsies was found to be associated with a poor response to treatment (*p* = 0.003, *p* = 0.026, *p* < 0.031, *p* < 0.001) [81,82,83,85]. Peng et al. also showed that COX-2 expression was an independent risk factor for the pCR after RCT [84]. Additionally, independent prognostic factors for the overall survival are age above the median, advanced pathological stage, tumor-positive resection margins and COX-2 expression. A correlation between COX-2 expression and preoperative treatment effects, as well as clinical prognosis, are demonstrated in Table 5, Table 6 and Table 7.

## 5. Discussion

During the last decades, therapeutic strategies for rectal cancer treatment have significantly evolved. The initial step that aimed to change the treatment guidelines regarding rectal cancer were two trials conducted by the National Surgical Adjuvant Breast and Bowel Protocol RO1 and Gastrointestinal Tumor Study Group Protocol 7175, which have established the beneficial impact of adjuvant radiation and chemoradiation therapy for local control and long-term survival. These results were confirmed by several subsequent prospective randomized trials.

The most important attribute of radio/radiochemotherapy is the ability to induce a complete pathological response, which equals the complete absence of tumor in surgical specimens. Despite the huge role of adjuvant treatment in decreasing local recurrence and improving overall survival, the results of treatment are varying from complete response to little or even no response. Usually, only 15–30% of patients show a favorable response to treatment with the usage of pCR. Thus, to quantify tumor response for neoadjuvant therapy, several techniques as well as numerous researches investigating different molecular mechanisms and molecules have been provided. In order to reduce a variation in tumor response, neoadjuvant regimens were also standardized. A significant number of patients have their surgical intervention delayed and those are unnecessarily exposed to the toxic effects of rectal cancer treatment that is usually ineffective, time-, and cost-consuming. The response to the combined neoadjuvant therapy predicts the final outcome. The clue task is to discover specific biomarkers or provide a method that could be potentially useful to differentiate the tumor ‘responders’ from the ‘non-responders’. The most important disadvantage of the neoadjuvant treatment is the fact that patients with a worse response cannot be identified until the time of pathologic analysis; all in all, the ‘non-responders’ (radio-resistance) are not selected for alternative treatment, which can improve their response. What is crucial is the histopathological assessment; however, imaging studies should also be provided in the case of rectal cancer—an MRI performed after radiotherapy aims to assess patients’ response to treatment and further qualification for potential surgery. At present, no means of predicting treatment response exists and all patients must undergo empirical preoperative treatment and surgery. Preoperative treatment is a well-suited model to evaluate and compare the expression of the biological factors (biomarkers are analyzed in the diagnostic biopsy and in the tumor specimen after treatment, correlation with an outcome). Currently, available therapeutic strategies are fundamentally based on the T and N stages and often do not consider a tumor’s biological genotype and phenotype [90,91]. This practice might result in either over-treatment or under-treatment. There is a need to identify a molecular biomarker that could be measured before preoperative treatment, allowing to divide patients into specific subgroups. Based on the observation that long-term use of a COX-2 inhibitor (rofecoxib) can reduce rectal polyp formation in patients with familial adenosis polyposis, COX-2 inhibitors are currently widely investigated [92,93]. Short-term usage of COX-2 inhibitors combined with preoperative radiochemotherapy turned out to be safe and effective for patients with locally advanced rectal cancer; however, more research in this matter is still needed [94]. The selective COX-2 inhibitor, used with radiation, can significantly increase tumor susceptibility to radiation by inhibiting the release of prostaglandins [95]. The first results of combined treatment with COX-2 inhibitor and neoadjuvant treatment are promising; efficacy and good toleration of celecoxib (COX-2 selective inhibitor) administration were observed [96,97].

This systematic review investigated a total of 2095 patients. The studies included in a final analysis were published between 2005 and 2020 and were focused on the analysis of the significance of COX-2 expression in rectal cancer preoperative radiotherapy or radiochemotherapy by predicting the response of given treatment. 80% of the studies were based on the retrospective analysis of COX-2 expression in pretreatment biopsy (paraffin blocks) compared to post-treatment/post-irradiation COX-2 expression. In each case, immunohistochemistry was used for COX-2 expression staining. In some of the studies, apart from COX-2 expression, the expression of other molecular markers was also investigated (p53, p21, p27, Bax, BCL-2, VEGF, APAF-1, CD34, Ki-67, VEGFR-2, EGFR, thymidine phosphorylase and others), which indicates the need of searching for other treatment predictors among molecular markers involved in cell growth, as well as in the prostanoid and apoptotic pathways. All of the presented methods of treatment were administrated according to the individual staging (TNM, Dukes’s classification) and in accordance with the ruling guidelines for rectal cancer treatment.

It should be considered that our study has some limitations that are mainly the consequences of the available literature. Although the systematic search was performed in The Cochrane Library, PubMed, Web of Science, and Scopus databases, only 13 researches were found. Each of the analyzed studies had a similar methodology but the study groups were not easy to collate due to the lack of important data (overall survival or summary of preoperative treatment) or lack of expression of COX-2 measurement details. Another difficulty was a standardization of given preoperative treatment—in four cases, preoperative radiotherapy was provided (in three research short-term radiotherapy—5 × 5 Gy, one research—long-term) and in four cases radiochemotherapy was administrated preoperatively. Some of the results seem to be inconclusive, probably due to the small study population. Further, it should be considered that COX-2 expression is slightly different interpreted amongst studies; the interpretation whether its expression was high or low was primarily based on the results of the ones studies rather than reference values, which de facto remain not constant as previously mentioned. Another limitation is the fact that the stages in which the samples were collected remained different between studies and further were sometimes not clearly defined by the authors.

Based on the systematic review, apart from investigating the role of COX-2 expression in neoadjuvant rectal cancer treatment response, we have proposed a novel pretreatment proceeding in rectal cancer, which is based on the well-established guidelines for rectal cancer treatment and the most recent research regarding the COX-2 inhibition during preoperative treatment (Diagram 1). Since it was observed that the increase of COX-2 activity amongst colorectal cancer patients is associated with greater progression of carcinogenesis, it is suggested that COX-2 expression might act as a prognostic factor in some aspects [98]. Further, COX-2 overexpression might induce further angiogenesis, which is also attributed to poorer clinical outcome, presenting its significance in clinical practice [99].

To the best of our knowledge, there are no accessible studies that summarize the effects of rectal cancer preoperative treatment in relation to the levels of COX-2 expression, which makes the presented study a novel review. Nevertheless, to confirm our results and validate the predictive power of COX-2 as a diagnostic marker, further additional independent research is needed.

## 6. Conclusions

A COX-2 expression is an early event involved in rectal cancer development. In cases with negative COX-2 expression, radiotherapy and radiochemotherapy may reduce the local recurrence. Taking those facts into consideration, COX-2 may be considered as a biological factor in selecting patients for more effective, less time-, and cost-consuming preoperative treatment. Administration of COX-2 inhibitors to patients with COX-2 overexpression, in an attempt to improve the response rate to neoadjuvant treatment, needs an assessment in further clinical trials.

## Figures and Tables

**Table 1:** Preoperative therapies most prevalently provided in Europe, United States and Canada.

Region	Preoperative Therapy Description
Europe	A short-course radiotherapy—1 week of radiation without chemotherapy (5 Gy × 5) followed by surgery the next week (TME < 10 days from the first radiation fraction.
United States and Canada	A long-course chemoradiotherapy—45–50.4 Gy, 1.8–2 Gy/fraction without or with 5-Fluorouracil (5-FU; bolus injections with leucovorin at 6–10 times during the radiation or continuous infusion or oral capecitabine), followed by radical surgery 6–8 weeks later

**Table 5:** Patients characteristics in the studied articles.

Ref.	Study	TNM Stage												Preoperative Treatment	Type of Resection				Effect of Preoperative Treatment (%)			
		0	I	II	III	IV	pT1	pT2	pT3	pT4	pN0	pN1-2	M	-	Hartmann	Rectal Amputation/Low Anterior	Abdominoperineal Resection	Unknown	Complete Tumor Regression (pCR)	Partial Regression	Absence of Tumor Regression	Overall Survival—OS
[75]	de Heer et al.	11	265	252	300	61								RTH	50	579	251	1				82% (at 24 months)
[78]	Bouzourene et al.						2	21	66	14				RTH		50	51	2	0%	79%	20%	Median—53 months
[76]	Pachkoria et al.													RTH		25	38					No data
[81]	Smith et al.						4	7	26	6	32	16		CRTH		39	10		10% (pCR) + 33% (near pCR)	22%	35%	No data
[77]	Giralt et al.							6	62	13	48	27		CRTH		48	33					Median—53 months
[83]	Edden et al.													CRTH		103	46		24.5% (pCR) + 15.1% (near pCR)	39.40%	21%	No data
[80]	Yeoh et al.													RTH								No data
[82]	Min et al.													CRTH	1	16			16,70%	50%	26.70%	No data
[87]	Shinto et al.						35		98		78	66		CRTH								
[79]	Wen et al.													RTH								
[84]	Peng et al.													Neo-CRTH					28%			
[86]	Sole et al.													Neo-CRTH								
[85]	Jafarian et al.													Neo-CRTH								

**Table 6:** COX-2 expression levels studies in the investigated articles.

Ref.	Study	COX-2 Expression Level	
[75]	De Heer et al. (2015)	**Irradiated Specimens:**	
		Absent	0.4%
		Weak	12.4%
		Moderate	59.2%
		Strong	28%
[78]	Bouzourene et al. (2008)	**Non-irradiated specimens:**	
		Absent	50%
		Weak	15.4%
		Moderate	15.4%
		Strong	19.2%
		**Irradiated specimens:**	
		Absent	11%
		Weak	44%
		Moderate	28%
		Strong	17%
[76]	Pachkoria et al. (2005)	**Non-irradiated specimens:**	
		Weak	22%
		Strong	51%
		**Irradiated specimens:**	
		Weak	18%
		Strong	53%
[82]	Min et al. (2008)	ND	
[81]	Smith et al. (2006)	**COX-2 overexpression**	**Tumor regression grade**
		0%	Complete
		10%	Moderate
		8%	Poor
		20%	Absent
[77]	Giralt et al. (2007)	**Irradiated specimens:**	
		Absent	48.6%
		Present	51.4%
[83]	Edden et al. (2010)	**Pretreatment biopsies:**	
		Weak	32.9%
		Moderate	34.9%
		Strong	32.2%
[80]	Yeoh et al. (2005)	ND	
[87]	Shinto et al. (2020)	**Irradiated specimens:**	
		**Retrospective cohort:**	
		Low	21.1%
		High	78.9%
		**Prospective cohort:**	
		Low	30.6%
		High	69.4%
[79]	Wen et al. (2020)	**Non-irradiated specimens:**	
		Absent	67.7%
		Present	32.3%
		**Irradiated specimens:**	
		Absent	52.2%
		Present	47.8%
[84]	Peng et al. (2016)	**Irradiated specimens:**	
		Low	58.5%
		High	41.5%
[86]	Sole et al. (2016)	ND	
[85]	Jafarian et al. (2016)	COX-2 expression was observed in 95.6% of cases with various extent and intensities.	

**Table 7:** Correlation between COX-2 expression and preoperative treatment effects and clinical prognosis.

Ref.	Study	COX-2 Expression versus Treatment Effects	COX-2 Expression versus Tumor Prognosis: - OS - DFS - Local/Distant Recurrence
[75]	De Heer et al. (2015)	High COX-2 expression is an independent poor prognostic factor for disease-free and overall survival in irradiated rectal cancer patients	(1) Significantly higher rate of distant recurrences (*p* = 0.005) in tumors with high levels of COX-2 expression after radiotherapy (2) In non-irradiated rectal cancer, COX-2 expression does not affect the local recurrence (*p* = 0.44), distant recurrences (*p* = 0.77), OS (*p* = 0.61) and DFS (*p* = 0.57) (3) In irradiated rectal cancer high levels of COX-2 expression associated with poor DFS (*p* = 0.004), poor OS (*p* = 0.006) (4) COX-2 expression significantly associated with decreased levels of apoptosis in irradiated tumors (*p* = 0.001)
[78]	Bouzourene et al. (2008)	(1) Inconclusive data (2) COX-2 is overexpressed in the majority of rectal cancer treated with radiotherapy and it plays a role in local relapse	(1) No significant correlation between the expression level of COX-2 and OS after radiotherapy but longer DFS among radiosensitive patients (2) High levels of COX-2 expression after radiotherapy associated with local recurrences (3) COX-2 expression correlates with enhanced tumor inflammation (*p* = 0.03) and tumor volume exceeding 30 cc (*p* = 0.05) (4) No correlation between COX-2 expression and clinicopathological features (gender, age, Duke’s stage, TNM, tumor regression)
[76]	Pachkoria et al. (2005)	COX-2 expression is an early event involved in rectal cancer development	(1) Weak COX-2 expression before and after radiotherapy is related to a lower rate of local recurrence (*p* = 0.02) (2) Radiotherapy may reduce local recurrence in cases with negative COX-2 expression (3) COX-2 expression elevated in primary tumors compared to normal mucosa; no difference between primary tumors and metastases (*p* = 0.38) (4) Duke’s stage not related to the effects of radiotherapy
[82]	Min et al. (2008)	COX-2 overexpression is a predictor of poor tumor regression	(1) COX-2 expression correlated more likely with poor response to treatment (*p* = 0.003) (2) In COX-2 overexpression tumors less histopathologic nodal downstaging (*p* = 0.03) (3) Any of patient with COX-2 overexpression attained complete regression of primary tumor after treatment
[81]	Smith et al. (2006)	COX-2 overexpression significantly associated with poor response to RCT	(4) Pretreatment biopsies with COX-2 overexpression demonstrate moderate or poor response to treatment (*p* = 0.026) (5) Low level of spontaneous apoptosis in pretreatment biopsies is associated with worse response to radiation (*p* = 0.0007)
[77]	Giralt et al. (2007)	Value of COX-2 as a biomarker is controversial	(1) No significant correlation between the expression level of COX-2 and OS and DFS after radiochemotherapy (2) No correlation between COX-2 expression and clinicopathological features (gender, age, Duke’s stage, number of tumors) (3) COX-2 expression does not predict treatment response
[83]	Edden et al. (2010)	Evaluation of pretreatment COX-2 expression may predict tumor response to neoadjuvant rectal cancer therapy	(1) COX-2 overexpression in pretreatment biopsies is related to poor tumor regression (*p* < 0.003) and less likelihood of T-downstaging (*p* < 0.03) after radiochemotherapy
[80]	Yeoh et al. (2005)		(1) No apparent difference in COX-2 expression between short-term radiation and long-term chemoradiation therapy
[87]	Shinto et al. (2020)	The expression of COX-2 was significant predictor of tumour response to preoperative RCT. However, expression levels of COX-2 showed no statistical significance.	(1) Low expression of COX-2 was independent or marginally independent parameter that influenced tumor regression grade (TRG). (2) TRG 3–4 was associated with positive IHC findings for reduced expression of COX-2 (*p* < 0.001).
[79]	Wen et al. (2020)	The expression of COX-2 had diagnostic value for rectal cancer patients preoperatively (the expression in biopsy sample was higher than that in surgical samples including distance normal mucosa (histologically free from tumor cells), *p* < 0.05)	(1) The patients with high COX-2 expression level in biopsy samples tended to have worse OS (*p* = 0.044 with RTH) and DFS, with or without radiotherapy. (2) Positive expression of COX-2 (RTH (*p* = 0.022) and nonRTH (*p* = 0.084) had reduced DFS time compared to those with negative tumors. (3) The expression of COX-2-RTH (*p* < 0.001) was independent prognostic factor for local/distance recurrence. Patients with high expression of COX-2 were more prone to suffer from tumor recurrence.
[84]	Peng et al. (2016)	Low expression of COX-2 was associated with achieving the highest pCR rate, which was significantly higher than those with high expression of COX-2.	(1) COX-2 expression was independent risk factor for pCR after neo-RCT.
[86]	Sole et al. (2016)	No significant differences in COX-2 expression level.	(1) No significant differences in COX-2 expression level.
[85]	Jafarian et al. (2016)	The mean COX-2 immunoreactivity extent in pre-RCT samples was significantly higher in cases with post-RCT biopsies showing >50% necrosis than those with <50% necrosis (*p* < 0.01)	(1) Patients with good response to neoadjuvant therapy did not show extensive COX-2 staining extent in pretreatment specimens. 90% of poor responders revealed extensive COX-2 staining extent (*p* < 0.001)

Abbreviations: RTH—radiotherapy, RCT—radiochemotherapy, OS—overall survival, DFS—disease free survival.
